# Gill structural change in response to turbidity has no effect on the oxygen uptake of a juvenile sparid fish

**DOI:** 10.1093/conphys/cow033

**Published:** 2016-08-26

**Authors:** H. Cumming, N. A. Herbert

**Affiliations:** Leigh Marine Laboratory, Institute of Marine Science, The University of Auckland, PO Box 349, Warkworth 0941, New Zealand

**Keywords:** Environmental stress, metabolism, suspended sediments, symmorphosis

## Abstract

Turbidity as a result of increased suspended sediments in coastal waters is an environmental stress of worldwide concern. Recent research on fish suggests that detrimental changes to gill structure can occur in turbid waters, with speculation that these alterations diminish fitness variables, such as growth and development, by negatively impacting the O_2_ uptake capacity (respiration) of fish. Specifically to address this unknown, the impact of turbid water on the gill structure, somatic growth rate and O_2_ uptake rates of a juvenile sparid species (*Pagrus auratus*) was addressed following exposure to five different turbidity treatments (<10, 20, 40, 60 or 80 nephelometric turbidity units) for 30 days. Significant gill structural change was apparent with a progressive increase in turbidity and was quantified as a reduction in lamellar density, as well as an increase in basal hyperplasia, epithelial lifting and increased oxygen diffusion distance across the lamellae. The weight of control fish did not change throughout the experiment, but all fish exposed to turbid waters lost weight, and weight loss increased with nephelometric turbidity units, confirming that long-term turbidity exposure is detrimental to growth productivity. The growth of fish could be impacted in a variety of ways, but the specific hypothesis that structural alteration of the gills impairs O_2_ uptake across the gills and limits growth fitness was not supported because there was no measurable difference in the standard metabolic rate, maximal metabolic rate, aerobic metabolic scope or critical oxygen saturation limit of fish measured in clear water after 30 days of exposure. Although impaired O_2_ uptake as a result of structurally adjusted gills is unlikely to be the cause of poor fish growth, the exact mechanism by which growth productivity is affected in turbid conditions remains unclear and warrants further investigation.

## Abstract (precis)

The effect of suspended sediments on the oxygen uptake of a sparid fish was examined. *Pagrus auratus* showed gill damage to suspended sediments over 30 days, as well as decreased somatic growth. However, gill damage as a result of turbidity exposure was not associated with a change in aerobic capacity.

## Introduction

The erosion of soil and its transportation to the marine environment is a natural process, but anthropogenic land-use changes are accelerating the rate and extent of sediment input to the coast as a result of deforestation, livestock farming, dairying, coastal developments etc. (Morrison *et al*., 2009). Coastal sedimentation is recognized as a major environmental stressor of worldwide concern (Gray, 1997; Airoldi, 2003; Thrush *et al*., 2004) and is arguably already causing significant degradation of aquatic ecosystems in New Zealand (Morrison *et al*., 2009). Although suspended sediment measures are an important parameter in environmental impact assessments throughout the world (Gray, 1997; Scarsbrook, 2008; Morrison *et al*., 2009), understanding the exact impacts of suspended sediments is crucial for effective ecosystem management.

Turbidity impacts many organisms (e.g. Airoldi, 2003; Morrison *et al*., 2009), but fish species are important to ecosystem management because they provide an important ecosystem service, they are vulnerable to environmental stressors and, in some cases, they are exploited commercially (Holmlund and Hammer, 1999; Worm *et al*., 2006). It is therefore important to document the direct and indirect impacts of turbid waters on fish populations. For example, freshwater fish exposed to elevated levels of suspended sediments show signs of gill damage following direct contact with sediments in the water, but indirect impacts are also apparently accrued in the form of low rates of feeding, reduced growth, delayed maturation and increased susceptibility to disease (Bruton, 1985; Newcombe and MacDonald, 1991; Sutherland and Meyer, 2007). There is, perhaps, less information available regarding the effects of suspended sediments on marine species, but the literature for these species is largely consistent with the effects seen in freshwater (above), albeit with different levels of sensitivity depending on the background level of exposure of the species (Au *et al*., 2004; Wong *et al*., 2013; Hess *et al*., 2015; Lowe *et al*., 2015). For example, clownfish ordinarily live in clear tropical waters, so exposure to relatively low levels of suspended sediment (45 mg l^−1^) incurs gill damage, with a significant increase in oxygen diffusion distance across the lamellae (Hess *et al*., 2015). In contrast, higher levels of turbidity are required to impact the gills of *Pagrus auratus* because of the turbid coastal waters they inhabit (Lowe *et al*., 2015).

Understanding the link between direct and indirect impacts of suspended sediments is important as it allows a full understanding of cause and effect and therefore the ecosystem changes likely to occur as result of increased sediment loads. In this regard, fish gills are very important to consider because they are in direct contact with the environment and are highly vulnerable because they present fragile and exposed membranes, vital for the transfer of gases, ions and nitrogenous compounds between the body and the environment (Evans *et al*., 2005). There is now clear evidence that structural changes to fish gills do occur as a result of increased suspended sediments (Au *et al*., 2004; Wong *et al*., 2013; Hess *et al*., 2015; Lowe *et al*., 2015). Morphological changes seen in gill structure as a result of suspended sediment exposure are thought to be a protective mechanism to protect the inner gill tissues (pillar system) from particulate abrasion (Mallatt, 1985). These changes are commonly observed in the form of epithelial hyperplasia (Hess *et al*., 2015; Lowe *et al*., 2015), lamellar fusion (Wong *et al*., 2013; Lowe *et al*., 2015), hyperplasia at the base of the lamella (Wong *et al*., 2013), epithelial lifting (Au *et al*., 2004; Wong *et al*., 2013) and hyperplasia of the pillar system (Au *et al*., 2004), all of which collectively increase the oxygen diffusion distance across the secondary lamellae (Hess *et al*., 2015). Although it would be logical to assume that altered gill structure (as above) would indirectly impact the efficiency of oxygen transfer from water to blood across the gills, this has not been established. For example, Wenger *et al*. (2014) observed a slow rate of development in clownfish larvae exposed to suspended sediments, and this was suggested by Hess *et al*. (2015) to be a consequence of respiratory stress caused by a drastic change in gill morphology. However, this conclusion was based only on evidence of gill damage, not measures of respiration *per se*. Likewise, Lowe *et al*. (2015) also presented evidence of an increase in ventilation with progressive turbidity in juvenile *P. auratus* but, even though this single measure cannot accurately signal a change in oxygen uptake, it was speculated that the respiratory impact of impaired gill function was associated with the poor growth performance of their fish. Likewise, the study of Wong *et al*. (2013) reported impacts on the respiration of juvenile *Epinephelus coicoides* as a result of sediment-induced gill damage but, again, although the term ‘respiration rate’ was used, changes in the transfer of oxygen across the gills were not documented (Wong *et al*., 2013). These are all entirely logical speculations, but it is now important to ascertain whether damage to the gill lamellae as a result of suspended sediment exposure does indeed reduce the capacity for oxygen transfer and lead to respiratory stress and reductions in performance as suggested (Wong *et al*., 2013; Hess *et al*., 2015; Lowe *et al*., 2015). This is an important direction for turbidity-related research because an adequate oxygen supply is vital in setting the limit to important bodily functions, such as growth (Claireaux and Lefrançois, 2007), reproduction (Holt and Jørgensen, 2015), intraspecific interactions (Biro and Stamps, 2010) and locomotion (Schurmann and Steffensen, 1994; Metcalfe *et al*., 2016). Oxygen uptake thus appears integral to the fitness of different organisms in different environments (Claireaux and Lefrançois, 2007; Biro and Stamps, 2010; Holt and Jørgensen, 2015; Metcalfe *et al*., 2016).

This study specifically set out to test whether structural gill damage as a result of turbidity exposure feasibly limits the O_2_ uptake capacity (respiration) of fish and, therefore, their somatic growth. A juvenile sparid fish species (the New Zealand snapper, *P. auratus*) was exposed to five different levels of water turbidity [<10, 20, 40, 60 and 80 nephelometric turbidity units (NTU)] over a 30 day period, after which their gill structure and various rates of oxygen consumption were examined. The NTU values employed in the present study are generally at the lower end of what *P. auratus* could experience in their natural habitat. Nephelometric turbidity unit records for the Hauraki Gulf from the Auckland Regional Council show that NTU values can be as high as 268 NTU for short periods, and Lowe *et al*. (2015) exposed snapper to turbid waters varying from <10 to 160 NTU because that ‘encompasses the natural range of juvenile snapper in the Mahurangi Harbour’. Established techniques in intermittent flow-through respirometry were used to compare the oxygen uptake rates of *P. auratus* across the five turbidity treatments. This involved measuring and comparing the standard metabolic rate (SMR), maximal metabolic rate (MMR) and aerobic metabolic scope (AMS) of fish in well-oxygenated conditions, in addition to the critical oxygen saturation (*S*_crit_) limit of fish in low O_2_ (i.e. hypoxic) conditions. Standard metabolic rate is defined as the minimal amount of oxygen required to support the aerobic metabolic rate of fish in a rested, post-absorptive state (Chabot *et al*., 2016). Maximal metabolic rate is defined herein as the maximal amount of O_2_ consumption following a forced chase event (Norin and Clark, 2016). Aerobic metabolic scope can then be calculated as the difference between MMR and SMR and represents the O_2_ exchange potential for non-essential processes, such as activity, growth and reproduction (Clark *et al*., 2013). Finally, *S*_crit_ is a valued measure of respiratory performance during exposure to low-O_2_ conditions because it represents the O_2_ saturation level at which the fish transitions from being an oxygen regulator to an oxygen conformer and can no longer maintain SMR (Schurmann and Steffensen, 1997). *Pagrus auratus* was selected as a model species for this study because any increase in suspended sediments is of utmost relevance to this recreationally and commercially fished species in New Zealand, given that it is abundant in the Hauraki Gulf where water turbidity is increasing at the highest rate (Morrison *et al*., 2009).

## Materials and methods

### Fish handling and treatment

Approximately 100 juvenile New Zealand snapper (*P. auratus*, Sparidae) ranging in weight from 15 to 60 g and with a fork length (FL) from 88 to 125 mm were sourced from the Plant and Food hatchery in Nelson and transported to the Leigh Marine Laboratory, where they were housed in three 500 litres flow-through black PVC seawater tanks at ambient sea temperature. Fish were held for a minimum of 4 weeks to allow recovery from any stress associated with transport and fed daily on commercial fish pellets (Economy floating fish pellets; Aqua One, P.R.C) at a rate of 3% body weight day^−1^. Prior to experimentation, fish were intraperitoneally implanted with PIT tags under anaesthesia (AQUI-S NZ Ltd, Lower Hutt, New Zealand). Fish were weighed before experimentation and again at the conclusion, allowing calculation of individual weight-specific growth rate (SGR, as a percentage of body weight per day), as follows:

$$\mathrm{SGR}\;=\ln m_{2}\;-\;\ln m_{1}/t_{2}\;-\;t_{1}\;\times \;100$$

where, *m*_1_ is the initial weight at the start of the growth period *t*_1_, and *m*_2_ is the final weight at the end of the growth period *t*_2_.

Measurements of turbidity were used in this study as a proxy for suspended sediment, and this was reported in NTU, which quantifies the scattering and absorption of light (Bruton, 1985). A closed-system method was used to keep sediment levels constant and continuously suspended over time in five 150 litre circular treatment tanks. This was achieved by pointing the outlet of two submersible pumps (EHEIM compact 600 and 300, Germany) 2 cm away from the bottom of the tanks so that water circulated down and around the tank and was held in continual motion. A circular bubbler tube was also laid around the bottom edge of each tank to provide aeration, but this also helped to prevent sediment settling out around the tank margins. Although sediment was not added to a non-turbidity control tank, this tank was equipped with the same array of pumps and bubblers.

Surficial (1 cm deep) estuarine sediment was collected from the Whangateau estuary (36°18′31.5″S, 174°46′46.9″E) to create turbidity. Sediment was wet sieved down to <63 µm and left to settle overnight. The clear top water was siphoned off, and sediment was refrigerated and stored. Sediment was added to the tanks as a slurry, and levels were adjusted by adding varying amounts of stock solution to achieve the intended turbidity level. Turbidity was monitored daily using a TSS portable hand-held measurement instrument (HACH, Germany), and small amounts of extra sediment were added if required. To ensure that water quality was maintained long term throughout the trial, the five tanks were cleaned and flushed every 3–5 days to prevent build-up of toxins, uneaten feed and faeces. Owing to the closed nature of the tanks, total ammonia was monitored daily; it never exceeded 6.0 mg l^−1^ and was usually <1.8 mg l^−1^. Water quality was therefore considered acceptable according to the results of Lemarie *et al*. (2004), and particularly because free ammonia, nitrate and nitrite concentrations were undetectable. Fish continued to be fed daily on pellets at 3% body weight day^−1^ throughout the whole experiment.

Five individual *P. auratus* were randomly assigned to each of the five treatment tanks and exposed to one of four turbidity treatment levels (20, 40, 60 or 80 NTU) or a control (<10 NTU) for 30 days. A maximum of five fish were added so that the fish density was low (2.3 kg m^−3^) and water quality as high as possible in the closed-system tanks. The addition of fish to the five treatment tanks was replicated temporally three times. The start time of each treatment was also staggered by 4 days to allow time for respirometry, ensuring that the duration of turbidity exposure was at least 30 days but no more than 34 days for each group. The NTU treatments were also randomly assigned to each tank between each replicate to safeguard against tank effects. At the start of each replicate, fish were randomly placed into turbidity treatment tanks at 18.0°C and allowed to acclimate to the tank for a period of at least 48 h. Fish assigned to the <10, 20, 40, 60 and 80 NTU treatments had starting weights of (mean ± SEM) 77.57 ± 6.82, 70.26 ± 7.79, 82.51 ± 6.22, 84.23 ± 6.63 and 81.63 ± 7.77 g, respectively, which were not significantly different from each other (one-way ANOVA, *F* = 0.325, *P* > 0.05). Pumps were then switched on for a second acclimation period of at least 48 h before sediment was added. Owing to the natural variation of seawater used in the closed-system tanks, the control tank was labelled <10 NTU, but almost all daily measures were <5 NTU.

### Respirometry

After fish had been exposed to turbidity for at least 30 days, measures of whole-animal oxygen consumption were obtained using automated intermittent flow-through respirometry. The SMR, MMR, AMS and *S*_crit_ limits were determined using the methodology of Cook and Herbert (2012), Cook *et al*. (2011) and Svendsen *et al*. (2016). Fish were placed into one of two custom-made respirometers that consisted of a chamber (2.4 or 2.59 litres) attached to a flush pump (EHEIM compact 600, Germany), and this entire apparatus was housed in a larger (100 litre) reservoir that was filled with fresh seawater filtered through a 5 µm filter. The respirometer chambers were filled with clear water, so turbidity was not maintained during this part of the experiment. Another external loop of tubing was also attached to each end of the chamber, and this contained: (i) an in-line pump (EHEIM compact 300, Germany) that continually mixed water in the chamber; and (ii) a cuvette that housed a fibre-optic oxygen dipping probe (OXROB; Pyroscience, Germany) so that oxygen in each chamber could be monitored. Water temperature was held constant at 18.0 ± 0.5°C. An extra pump (EHEIM 600; Eheim, Germany) was also used to circulate seawater between the 100 litre reservoir and a 40 litre oxygenation/deoxygenation gas tower at a rate of 600 l h^−1^. Oxygen saturation in the reservoir and respirometry chambers was therefore controlled by bubbling air (for oxygenation) or compressed nitrogen (for deoxygenation) through a bubbler at the bottom of the gas tower. To limit bacterial respiration, seawater circulating between the reservoir and the gas tower was passed through an ultraviolet sterilizer (PondOne ClearTec, China). A blackout sheet shrouded the whole set-up and was designed to limit external disturbance.

After individual fish were sealed in each of the respirometry chambers, a repeating cycle of flush (2–3 min), wait (30 s) and measure (6–9 min) was initiated by customized software operating on a PC notebook via a relay control unit (USB Net Power 8800 Pro; Aviosys, Taiwan). The decline in chamber oxygen was recorded by the fibre-optic oxygen dipping probe coupled to a Firesting oxygen meter (Pyroscience, Germany) and used to calculate the mass-specific rate of oxygen consumption ($\dot{M}O_{2}$, in milligrams of oxygen per kilogram per hour) according to the following equation:

$$\dot{M}O_{2}\;=\;V\left(\Delta \%sat/t\right)\alpha .M_{Β}$$

where *V* is the respirometry chamber minus fish volume, Δ%sat/t is the change in oxygen saturation per unit time, α is the solubility coefficient of oxygen (in milligrams of oxygen per percentage level of O_2_ saturation per litre) in water (35 ppt, 18°C), and *M*_B_ is the body mass of the fish in kilograms (Schurmann and Steffensen, 1997). Fish were transferred in water and introduced to the chambers within 1 min at ~16.00 h each day and left undisturbed for ~16 h until the next day. In the region of 70–150 $\dot{M}O_{2}$ cycles were therefore collected from each individual overnight when water was fully air saturated, and these data were used to calculate the SMR of each fish in normoxic conditions using the 15% quantile method of Dupont-Prinet *et al*. (2010).

Once SMR was resolved, the *S*_crit_ of fish was assessed by gradually decreasing chamber oxygen in a stepwise fashion (70, 60, 50, 40, 30 25, 20 and 15% oxygen saturation) until a clear ‘break’ in $\dot{M}O_{2}$ from SMR was observed. The *S*_crit_ was calculated as individual *S*_crit_ and as overall *S*_crit_. Individual *S*_crit_ was resolved using the methodology of Schurmann and Steffensen (1997), where individual $\dot{M}O_{2}$ values below SMR were used to construct a linear regression of $\dot{M}O_{2}$ against O_2_ saturation (with a forced *y* intercept of zero). The O_2_ saturation level at which the regression intercepted with SMR was then taken as the *S*_crit_ level of each individual. Individual *S*_crit_ was then averaged and compared statistically between the five NTU treatments. To validate individual *S*_crit_, overall *S*_crit_ was also calculated using the methodology of Cook and Herbert (2012). This method used a one-way repeated-measures ANOVA to test the null hypothesis that average $\dot{M}O_{2}$ values under declining oxygen were not significantly less than mean SMR in normoxic conditions. A *post hoc* test identified $\dot{M}O_{2}$ values that failed this hypothesis, and these data were included in a linear regression, with a forced *y* intercept of zero. The SMR was extrapolated across the entire range of water oxygen saturation, and the associated point of intercept between the two regressions was taken as overall *S*_crit_.

Once both measures of *S*_crit_ were resolved, fish were returned to their respective treatment tank for 2–5 days. The MMR of fish was then determined using the exhaustive chase protocol of Cook *et al*. (2011) (see also review by Norin and Clark, 2016), where fish were manually chased with tail taps in a circular tank to the point of exhaustion for 5 min. Fish were then transferred immediately to the respirometer, and MMR was taken as the highest of at least three $\dot{M}O_{2}$ values.

Aerobic metabolic scope was calculated as the difference between MMR and SMR (Schurmann and Steffensen, 1997). After MMR and AMS calculation, fish were removed from the chamber, euthanized and their length and weight recorded.

Fish weight varied from 26.2 to 132 g; therefore, to account for any potential body mass scaling effects in the data, all $\dot{M}O_{2}$ values were standardized (corrected) to that of a 70 g fish using the following equation:

$$\dot{M}O_{2(70g)}\;=\;\dot{M}O_{2(\mathrm{meas})}{\left(\frac{w}{w_{\left(70g\right)}}\right)}^{\left(1-A\right)}$$

where $\dot{M}O_{2(\text{70g})}$ is the $\dot{M}O_{2}$ for a fish with the standardized (corrected) new weight of 70 g, $\dot{M}O_{2(\text{meas})}$ is the measured $\dot{M}O_{2}$, *w* is the weight of the fish, *w*_(70g)_ is the standardized body weight of fish set to 70 g and *A* is the weight exponent describing the relationship between metabolic rate and body weight. A mass scaling exponent of *A* = 0.8 was used (Skov *et al*., 2011, 2015).

### Histological analysis of gill tissues

After euthanasia and length and weight measurements, the first left gill arch of each fish was dissected out and fixed in Bouin's solution for 48 h, then transferred to 70% ethanol. Gill sectioning and histological preparation were carried out by Gribble Veterinary Pathology services (Mt Wellington, Auckland, New Zealand) where tissues were dehydrated through a series of graded ethanol concentrations (70, 95 and 100%) and embedded in a mould to form tissue blocks. Microtomy was then performed at 3 µm to produce gill sections. These sections were mounted on glass slides and stained with haematoxylin and eosin. Samples were then examined under a microscope (Leica DMRE, Wetzlar, Germany) equipped with a colour video camera (Leica DC500, Heerbrugg, Switzerland). One image from each sample was taken at a magnification of ×2.5 to measure the density of secondary lamellae (termed ‘lamellae’ hereafter). Three to five filament sections were then randomly selected and photographed at ×40 magnification. At ×40 magnification, four lamellae (two on each side of the filament) were measured in terms of epithelial thickness, epithelial lifting, oxygen diffusion distance and basal hyperplasia. These analyses were carried out using SigmaScan Pro 5, based on measurements used by Au *et al*. (2004) and Hess *et al*. (2015), and were carried out blinded with respect to treatments.

At ×2.5 magnification, lamellar density was calculated as the number of lamellae per micrometre of filament length, based on an average of three data values from each fish in each treatment group. For measurements at ×40 magnification, it is important to note that a lamella comprises a number of different tissues, including the outer epithelial tissue region (ET) and the inner pillar system (PS). Between these two tissues, there can be non-tissue space (NT). These tissues added together make up the lamellar area (L). The thickness of the epithelium (epithelial thickness) was calculated as the percentage area of epithelial tissue (ET/L). Epithelial lifting was calculated as the percentage area of non-tissue space (NT/L) (however, it should be noted that not all measures revealed signs of epithelial lifting; see ‘*Statistical analysis*’ section below). To calculate the oxygen diffusion distance, the area of the pillar system (PS) was subtracted from the area of the functional lamella (L), then divided twice by the length of the functional lamella to obtain the oxygen diffusion distance. In order to assess basal hyperplasia, the thickness of the filament (in micrometres) was measured from the epithelial edge of the filament to the filament mid-line. This quantifies the addition of cells on the filament at the base of lamellae, which may thicken until two lamellae are completely fused (Wong *et al*., 2013). Measures for each treatment were taken from at least eight to 10 fish and from between 74 and 119 lamellae.

### Statistical analysis

If the assumptions of normality and homoscedasticity were satisfied, individual one-way ANOVAs were used to test the effect of the turbidity treatment (control, 20, 40, 60 and 80 NTU) on each of the respiratory (SMR, MMR, AMS and *S*_crit_) and gill morphometric variables (lamellar density, basal hyperplasia, epithelial thickness, epithelial lifting and oxygen diffusion distance). There was no observable difference between the three temporal periods for any measure, so the data for each turbidity treatment were pooled. Where the effect of turbidity treatment was found to be positive, specific pairwise comparisons between the five treatments were carried out using a Holm–Sidak *post hoc* test. Where test assumptions could not be satisfied, a Kruskal–Wallis one-way ANOVA on ranks was performed with Dunn's *post hoc* test for specific pairwise comparisons. Statistical comparisons could not be attempted on data showing the total average of epithelial lifting between treatments because epithelial lifting was not evident in all samples. Therefore, to show the true extent of epithelial lifting when present, data relating to no epithelial lifting were excluded, and the remaining data were replotted and subjected to statistical testing as above. Significance was accepted at *P* < 0.05 in all cases, and all analyses were performed in Systat Software Inc., USA.

## Results

### Gill structural change

**Figure 1:**
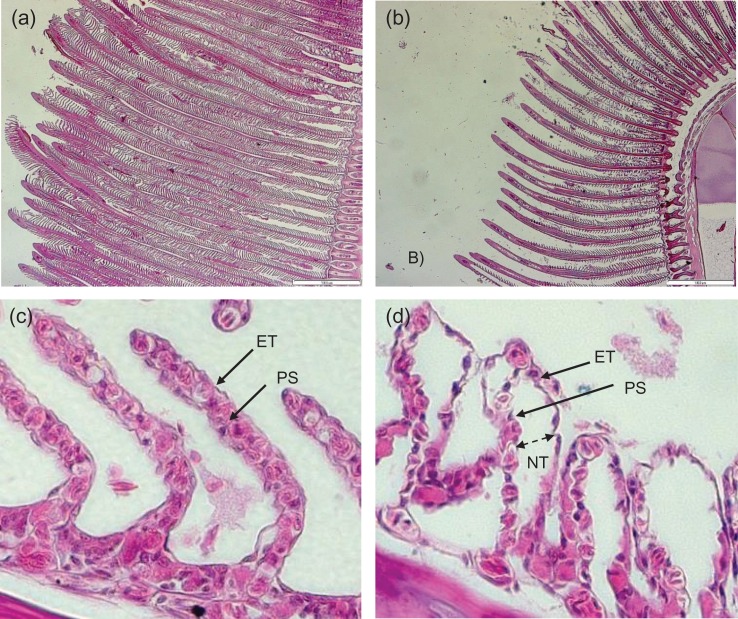
Light micrographs of gills from *Pagrus auratus*. (**a**) Control lamellae at ×2.5 magnification. Filaments are relatively uniform in length, and lamellae are dense and mostly intact. (**b**) Lamellae exposed to 80 nephelometric turbidity units (NTU) at ×2.5 magnification. Filaments are not of uniform length, and many lamellae appear to be missing or fused. (**c**) Control lamellae at ×40 magnification showing epithelial tissue (ET) region, the pillar system (PS). Non-tissue space within the epithelial tissue is not conspicuous. (**d**) Lamellae exposed to 80 NTU at ×40 magnification. Non-tissue space (NT) underneath the epithelial region is obvious, leading to epithelial lifting.

**Figure 2:**
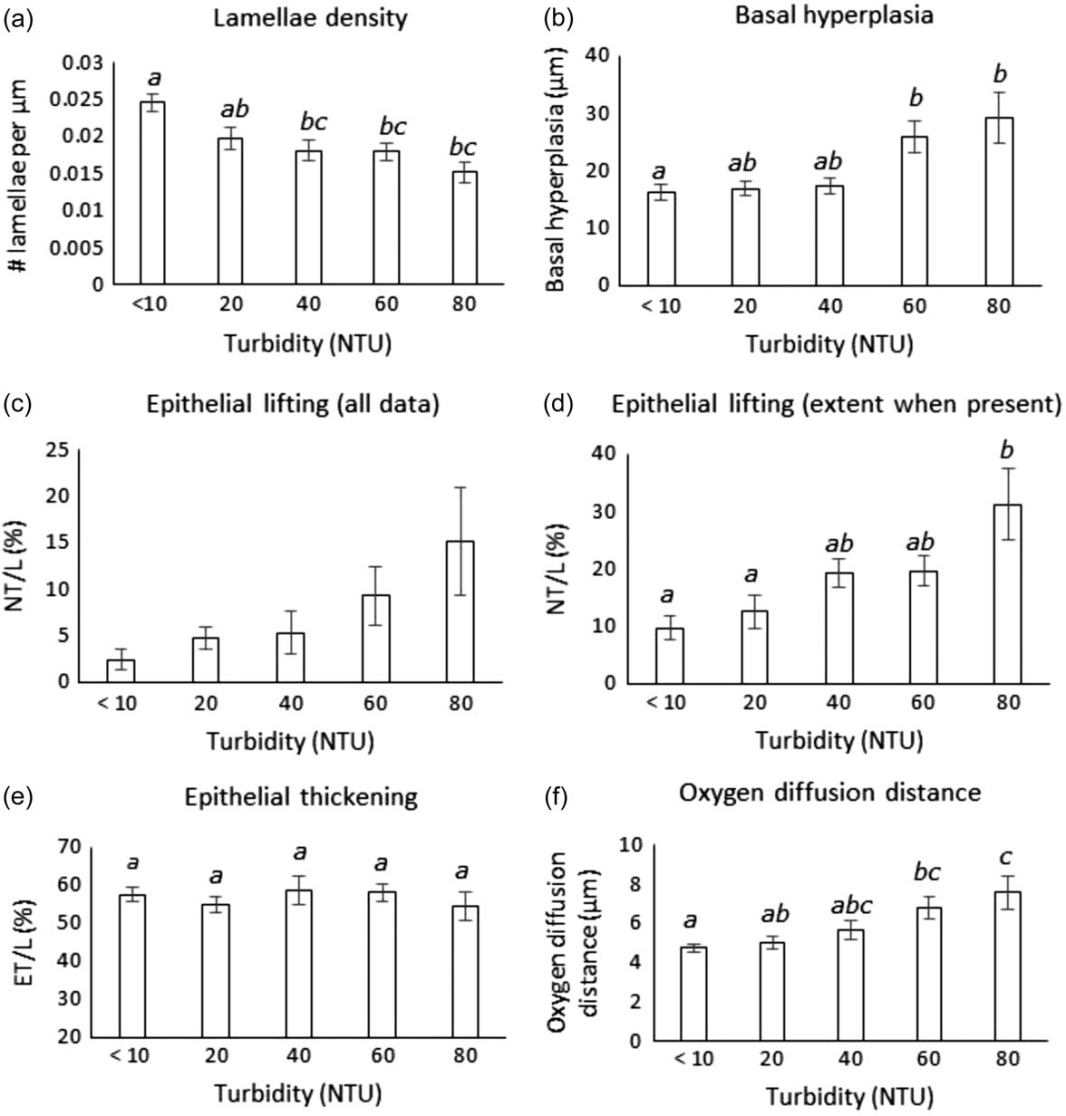
Morphometric measures (mean ± 95% confidence interval) of *P. auratus* secondary lamellae exposed to the five turbidity treatments (<10, 20, 40, 60 and 80 NTU). (**a**) Secondary lamellar density expressed as the number of lamellae per micrometre length of gill filament. (**b**) Hyperplasia at the base of lamellae. (**c**) Average epithelial lifting (including no lifting), represented by the percentage of the lamella area (L) occupied by non-tissue space (NT). (**d**) The extent of epithelial lifting where present, represented by the percentage of the lamella area (L) occupied by non-tissue space (NT). Data not showing epithelial lifting are excluded. (**e**) Epithelial thickness represented by the percentage of the lamella area (L) occupied by epithelial tissue (ET). (**f**) Oxygen diffusion distance. Bars with different letters are significantly different (*P* < 0.05).

Turbidity was found to exert a highly significant effect on the density of lamellae on the gills (one-way ANOVA, *F* = 7.66, d.f. = 4, *P* < 0.01). Control group lamellae were mostly intact and of equal length (Fig. 1a), but at 80 NTU lamellae appeared to be missing, fused, shorter or of non-uniform length (Fig. 1b). *Post hoc* tests confirmed this trend, because there were no significant differences in lamellar density at 40, 60 and 80 NTU with respect to the control treatment (*P* < 0.05; Fig. 2a).

#### Basal hyperplasia

There was a highly significant turbidity treatment difference in terms of basal hyperplasia (Kruskal–Wallis ANOVA, *H* = 14.27, d.f. = 4, *P* < 0.01), which increased with increasing turbidity, with significant differences observed between at 60 and 80 NTU with respect to the control (*P* < 0.05; Fig. 2b).

#### Epithelial lifting

Of the gill samples that showed epithelial lifting, turbidity was found to have a highly significant effect on the extent of lifting (one-way ANOVA, *F* = 5.62, d.f. = 4, *P* < 0.01). Epithelial lifting was significantly different between the 80 NTU group and the control group (*P* < 0.05; Figs 1c and 2d).

#### Thickness of epithelium

Turbidity was found to have no effect on epithelial thickness (one-way ANOVA, *F* = 0.42, d.f. = 4, *P* > 0.05; Fig. 2e).

#### Oxygen diffusion distance

There were highly significant turbidity treatment differences in terms of oxygen diffusion distance (Kruskal–Wallis ANOVA, *H* = 20.64, d.f. = 4, *P* < 0.01). Oxygen diffusion distance increased progressively with increasing turbidity treatment, with significant increases observed at 60 and 80 NTU with respect to the control group (*P* < 0.05; Fig. 2f).

### Respirometry

**Figure 3:**
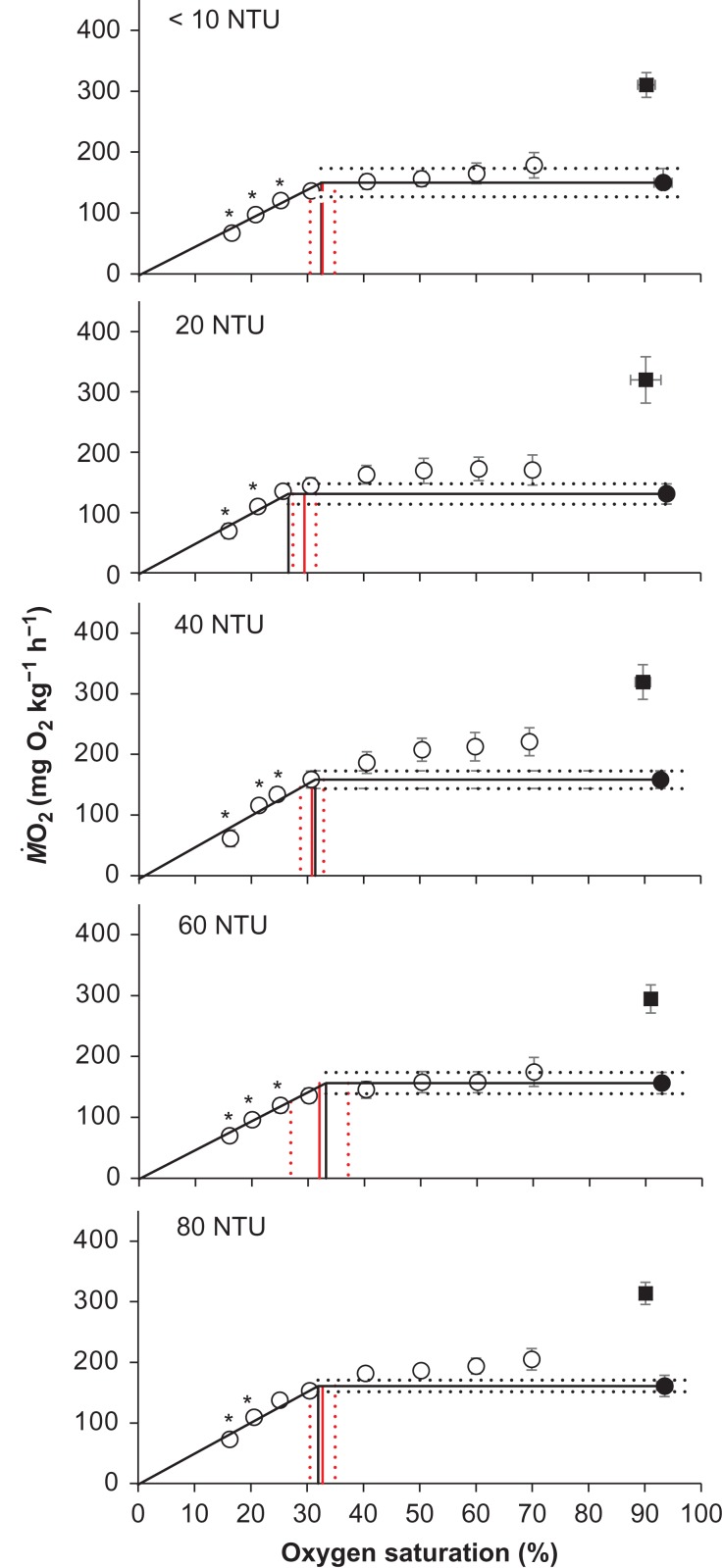
The whole-animal O_2_ consumption rates ($\dot{M}O_{2}$) of *P. auratus* exposed to five turbidity treatments (<10, 20, 40, 60 and 80 NTU) at 18°C; *n* = 14, 9, 9, 10 and 11 for each treatment group, respectively. The filled circle identifies standard metabolic rate (SMR) with normoxic oxygen saturation. Mean SMR was extrapolated across the range of oxygen saturation levels investigated (continuous horizontal line) with associated error values indicated (dotted horizontal lines). The filled square indicates mean maximal metabolic rate (MMR) values with normoxic oxygen saturation. Open circles indicate mean $\dot{M}O_{2}$ values during the progressive reduction in water oxygen saturation. The $\dot{M}O_{2}$ values that were found to be significantly below SMR are denoted with an asterisk. A sloping regression is plotted through these values with a forced *y* intercept of 0. The point of intercept between this regression line and the extrapolated mean value of SMR indicates the point of overall critical oxygen saturation (*S*_crit_) and is denoted by a thin vertical black line. Mean individual *S*_crit_ is also shown with a continuous vertical red line and 95% confidence limits as vertical dotted red lines. See the Materials and methods section for more detail regarding *S*_crit_ calculations. All symbols and error bars represent mean values ± 95% confidence intervals.

Turbidity had no effect on SMR (*F* = 1.28, d.f. = 4, *P* > 0.05), MMR (*F* = 0.78, d.f. = 4, *P* > 0.05), AMS (*F* = 1.38, d.f. = 4, *P* > 0.05) or *S*_crit_ (*F* = 1.06, d.f. = 4, *P* > 0.05). There was therefore no statistically significant difference between treatment groups for any of the metabolic parameters measured (Fig. 3). Overall *S*_crit_ measures were generally within the measurable range of individual *S*_crit_ measures at each NTU level (Fig. 3), except perhaps at 20 NTU, where overall *S*_crit_ (26.6% O_2_ saturation) appeared slightly lower than the rest of the 20 NTU treatments (27.4–31.5% O_2_ saturation).

### Growth

**Figure 4:**
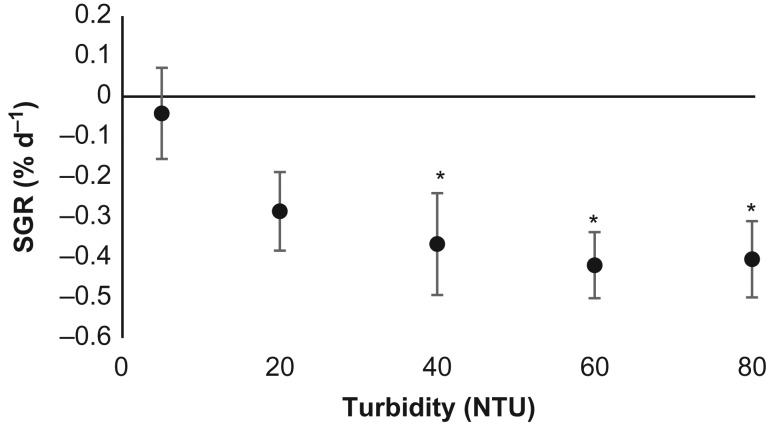
The mean weight-specific growth rate (SGR) of *P. auratus* exposed to the five turbidity treatments (<10, 20, 40, 60 and 80 NTU) for 30 days. Data are presented as mean values ± 95% confidence intervals. *Significant difference from the control (*P* < 0.05).

Turbidity had a highly significant effect on fish SGR over 30 days of NTU exposure (Kruskal–Wallis ANOVA, *H* = 25.76, d.f. = 4, *P* < 0.01). The control <10 NTU group maintained weight, but there was a significant loss of weight observed at 80, 60 and 40 NTU with respect to the control group (*P* < 0.05; Fig. 4).

## Discussion

Anthropogenic activities play a major role in increasing the rate of sediment run-off from land that renders coastal environments turbid (Morrison *et al*., 2009). This worldwide phenomenon appears to be escalating, but managing the problem might be facilitated if it is more precisely understood how turbid waters affect marine biota. There are several reports of fish suffering gill damage as a result of exposure to suspended sediment (Wong *et al*., 2013; Hess *et al*., 2015; Lowe *et al*., 2015), and these fish also appear to show slower rates of growth or longer rates of development and even mortality (Wenger *et al*., 2012; Wong *et al*., 2013; Lowe *et al*., 2015). The authors of these studies have logically speculated that oxygen transfer across damaged gills must be sufficiently impaired that fish experience a long-term cost with respect to individual fitness performance. However, being the first investigation of the aerobic physiology of fish from turbid waters, the present study does not wholly support this hypothesis. Juvenile *P. auratus* that were exposed to increasingly turbid conditions did indeed show significant gill structural change and weight loss, as seen in previous studies (Figs 1, 2 and 4; Lowe *et al*., 2015), but there was no measurable change in any measure of oxygen uptake in well-oxygenated conditions (normoxia), and fish could even maintain the same rate of oxygen uptake during a low-oxygen challenge (hypoxia) test (Fig. 3). It should be noted, however, that the present study measured the O_2_ uptake of gill-damaged fish only in non-turbid conditions. These experiments cannot, therefore, fully exclude the possibility that turbidity impairs O_2_ uptake via an alternative pathway, such as gill clogging (see discussion below in the ‘*Growth productivity and fitness in turbid waters*’ section).

### Gill structural response to turbid water

Significant gill structural change in response to increasingly turbid waters was evident in *P. auratus* and was characterized not only by a measurable decrease in gas exchange surface area but also by an increase in gas diffusion distance at the level of the secondary lamellae. Loss of secondary lamellae is considered to be the direct net effect of sediment particle abrasion (Lake and Hinch, 1999), and individual *P. auratus* showed a decrease in lamellar density on the first gill arch with increasing levels of turbidity (Fig. 2a). If assumed to be a true representation of change across the whole gill, this observation alone should indicate that the oxygen uptake potential of *P. auratus* in turbid treatments is limited by a significant reduction in functional gill surface area. Likewise, the observed increase in basal hyperplasia with increasing turbidity (Fig. 2b; which is thought to precede complete lamellar fusion) would also be expected to reduce the gill surface area for oxygen exchange further.

*Pagrus auratus* exposed to increasingly high-NTU treatments showed a progressive increase in oxygen diffusion distance across the secondary lamellae that was driven entirely by epithelial lifting (Fig. 2c and d) rather than by epithelial thickening (Fig. 2e). This is presumably a response to protect the inner pillar cell system from the abrasive action of suspended sediment and to prevent irritants from diffusing into the bloodstream (Mallatt, 1985). Au *et al*. (2004) also found evidence of epithelial lifting in juvenile green grouper (*Epinephelus coioides*) exposed to ≥200 mg l^−1^ (equivalent to >80 NTU) compared with non-suspended sediment controls. Likewise, Wong *et al*. (2013) documented evidence of epithelial lifting and hyperplasia from the base of the lamellae exposed to suspended sediments. Another related study by Hess *et al*. (2015) showed that tropical clownfish larvae exposed to suspended sediments experienced a 56% increase in oxygen diffusion distance, but this difference was the result of hyperplasia of the epithelial tissue (Hess *et al*., 2015). Epithelial hyperplasia was also observed with increasing turbidity in a study by Lowe *et al*. (2015). Although these studies did not necessarily assess the same parameters nor obtain the same results as the present study, one consistent feature is that they all show structural changes synonymous with an increase in the diffusion distance of the lamellae. Not surprisingly, some authors speculated that this may reduce the efficiency of oxygen uptake across the gills, which could result in a reduction of oxygen transport to the organs, causing respiratory stress (Au *et al*., 2004; Wong *et al*., 2013; Hess *et al*., 2015; Lowe *et al*., 2015). In support of such speculation, studies by Bindon and colleagues (Bindon *et al*., 1994a,b) also suggest that structural change brought about by chloride cell proliferation on the secondary lamellae can negatively impact blood oxygen saturation, hence O_2_ uptake potential across the gill.

### Rates of O_2_ consumption in response to turbid water

The present study set out to resolve whether gill structural change via turbidity exposure would reduce the capacity for oxygen transfer but, despite evidence of major gill restructuring, no significant change in oxygen uptake was found with increasing turbidity. All metabolic measures of SMR, MMR and AMS as well as *S*_crit_ breakpoints were comparable between the five turbidity treatments (including the control), with no marked differences between any of the groups. It is feasible that compensatory mechanisms were able to maintain (i.e. steady) the SMR of fish with structurally modified gills, but it is far harder to understand why MMR, AMS and *S*_crit_ were all unaffected in fish impacted by turbidity. To explain why fish in this study showed no change in SMR, either (i) the structurally modified gills of turbidity-exposed fish were not functionally impaired in terms of O_2_ uptake or (ii) a range of physiological adjustments compensated for reduced oxygen transference across the gills so that basal and/or routine metabolic function (SMR) was maintained. In support of the latter possibility, Dussault *et al*. (2001) discovered that structural changes to the gills of rainbow trout as a result of aluminium exposure led to an increase in blood haematocrit and blood haemoglobin concentration. In more severely affected fish, the increase in haematocrit and haemoglobin concentration was also associated with an increase in heart rate and stroke volume (Dussault *et al*., 2001). In another study of interest, Gold *et al*. (2015) showed that an increase in cardiac output was sufficient to maintain routine oxygen consumption in anaemic rainbow trout that had reduced aerobic capacity (Gold *et al*., 2015). Only one study to date has specifically examined the aerobic physiology of fish in response to turbidity (Lowe *et al*., 2015) and, in that study, it appeared that the gill ventilation rate of *P. auratus* was increased in turbid waters, but how this contributed to oxygen uptake across structurally modified gills was unfortunately not ascertained. It is possible that the observed increased in the rate of ventilation witnessed by Lowe *et al*. (2015) allowed *P. auratus* to maintain core body function (i.e. SMR) with structurally modified gills, but it is also equally feasible that the ventilatory response of snapper observed in turbid water was a direct response to gill clogging, not modified gill structure *per se*.

Although SMR might be held constant through a plethora of compensatory mechanisms, it is far harder to understand how MMR, AMS and *S*_crit_ (as indicators of respiratory performance under challenge) were held steady with structurally modified gills, as seen in the present study. There is simply no evidence that turbidity affects the oxygen extraction capacity or efficiency of this species. Although contradictory to the main hypothesis of the study and that of Hess *et al*. (2015) and Lowe *et al*. (2015), the fact that high-NTU treatments maintained MMR, AMS and *S*_crit_ with apparently damaged (or at least highly modified) gills serves to challenge the concept of symmorphosis. Symmorphosis states that biological design is optimized and that structure matches functional requirements with no excess provisions (Taylor and Weibel, 1981), but the results of the present study do not support this theory because the oxygen uptake rates of fish in the higher NTU treatments were exactly the same as those of the <10 NTU control fish, despite the presence of fewer secondary lamellae with increased oxygen diffusion distance. It is therefore concluded that <10 NTU controls do not show an optimized design but instead have gills furnished with secondary lamellae and thin epithelia that are potentially in excess of functional requirements. A number of other studies also present evidence of ‘excessive construction’ that refute the concept of symmorphosis (Garland and Huey, 1987; Chappell *et al*., 2007) but, as argued by Randall and Brauner (1991), ‘structures are designed to satisfy functional requirements for operation over a wide range of conditions, rather than optimally for a given set of conditions’. Therefore, *P. auratus* may possibly maintain reserve gill capacity in non-turbid conditions as an evolutionary safeguard against diminished respiratory performance at times of environmental stress (e.g. increased turbidity). The loss of lamellar surface area may not therefore result in the loss of respiratory performance, as evidenced by *P. auratus* with their maintained rates of metabolism. Studies on the crucian carp, *Carrassius auratus*, may reinforce this view because this species maintains routine metabolic function in well-oxygenated conditions with simple gills that lack protruding lamellae (Sollid *et al*., 2003).

### Growth productivity and fitness in turbid waters

With respect to the growth of fish in turbid waters, this study is consistent with the observations of others, because increasing turbidity was shown to impair fish growth productivity. Indeed, all fish in turbidity treatments except the <10 NTU control group lost significant weight, and the greatest weight loss was witnessed in the highest turbidity group. Any expectation that fish would show positive weight gain in this study is probably unrealistic for an experiment of this nature because of the following factors: (i) the turbulent nature of water in the tanks that held turbidity treatments constant; (ii) the regular disturbance and handling of fish so that water quality was maintained; and (iii) the use of 18°C water, which is a few degrees below the optimal growing temperature of *P. auratus* (Francis, 1997; Fielder *et al*., 2002). As such, it is encouraging that fish in the <10 NTU treatment maintained their weight throughout the 30 day experiment, especially as the equivalent control treatment in the study of Lowe *et al*. (2015) lost significant weight. Although the wild-caught snapper of Lowe *et al*. (2015) should have been more accustomed to turbid waters in their natural habitat, the hatchery-reared fish of the present study were probably more tolerant of laboratory conditions, which may reflect the difference in growth performance seen between the two studies.

The important question posed by the speculative discussion of Hess *et al*. (2015) and Lowe *et al*. (2015) is whether the fitness consequence of growth loss is the direct result of impaired oxygen transfer across the gills. The present study can provide an answer to this question because, although growth fitness was clearly impacted by turbidity, no observable change in any metabolic rate of gill-damaged fish was observed, suggesting that poor growth is not likely to be the result of impaired O_2_ uptake brought about by gill structural change. This study, in which the respiration of fish was measured in non-turbid clear water, cannot exclude the possibility that turbidity impairs O_2_ uptake via direct gill clogging and that growth was impacted by this alternative pathway. In order to answer this question, future studies will need to undertake the technically challenging task of measuring the O_2_ uptake rate of fish in turbid conditions in respirometers. If respiratory stress of any form is still not found to be the cause of poor growth in turbid conditions, researchers should then, perhaps, consider the possibility that other factors are involved, such as the simple loss of visual performance (Meager and Batty, 2007; Johansen and Jones, 2013), impaired osmoregulation (Au *et al*., 2004; Wong *et al*., 2013) or nitrogenous waste excretion across the gills (Randall and Wright, 1987).

### Conclusion

Our results demonstrate that fish productivity as a potential fitness measure of fish in and around the Hauraki Gulf is likely to be impacted by rising turbidity levels. However, contrary to previous speculation (e.g. Hess *et al*., 2015; Lowe *et al*., 2015), it is unlikely to be the result of oxygen transfer deficits across structurally adjusted gills, because *P. auratus* was able to maintain all rates of metabolism in clear water after turbidity exposures. Diminished O_2_ transfer across gills clogged with sediment could still be involved, so future research still needs to identify the exact mechanism that underlies the poor growth observed, but the take-home message of the present study is not entirely dire. If increased turbidity remained an episodic event driven by infrequent storm events, the fact that *P. auratus* are not oxygen limited as a result of gill structural change exemplifies that the aerobic physiology of this species may be more resilient to environmental change than previously assumed. The finding that gill structure is possibility maintained in excess of functional requirements opposes the theory of symmorphosis but suggests that the anatomy of some fish species may have capacity to buffer oxygen uptake against the harmful effects of environmental change (Randall and Brauner, 1991). This resilience is likely to be of particular importance to species such as *P. auratus* that are subject to both commercial and recreational fishing pressure in a rapidly changing world.
